# Autoimmune Disease Flare-Up in a Case of Locally Advanced Breast Carcinoma Controlled by Surgical Resection

**DOI:** 10.7759/cureus.96544

**Published:** 2025-11-11

**Authors:** Ilango Parthasarathy, Gowtham Karthik V, Syed A Hussain

**Affiliations:** 1 Surgical Oncology, Sri Ramachandra Institute of Higher Education and Research, Chennai, IND

**Keywords:** autoimmune disease, breast carcinoma, ductal carcinoma immunomodulation, invasive paraneoplastic syndrome, lupus erythematosus, reactive oxygen species (ros), regulatory t cells (tregs), surgical resection, tumor microenvironment

## Abstract

Autoimmune diseases and malignancies share complex immunological interactions, but their coexistence in a single patient presents unique diagnostic and therapeutic challenges. We report the case of a 65-year-old woman with a history of autoimmune disease who presented with a locally advanced carcinoma of the breast. Despite ongoing immunosuppressive therapy, she developed a flare of autoimmune manifestations during the progression of her malignancy. The patient underwent surgical resection of the primary tumor, which not only achieved local oncological control but also resulted in a marked improvement of her autoimmune symptoms postoperatively.

This case highlights the dual nature of the immune system in cancer, where immune dysregulation contributes both to tumor progression and to autoimmunity. The clinical course of our patient suggests that tumor antigens may serve as a chronic stimulus for immune activation, thereby exacerbating autoimmune flares. Surgical resection eliminated the source of antigenic stimulation, leading to symptom resolution, underscoring the potential therapeutic role of surgery beyond oncological benefit. Recognition of autoimmune disease exacerbations as a manifestation of underlying malignancy is crucial, as they may mimic paraneoplastic syndromes and delay appropriate treatment. A multidisciplinary approach involving oncologists, surgeons, and rheumatologists is essential for optimal care in such complex cases. This report adds to the limited literature on autoimmune disease flares associated with breast carcinoma and suggests that timely surgical intervention can achieve disease control on two fronts, cancer progression and immune dysregulation.

## Introduction

Autoimmune diseases are chronic disorders caused by dysregulation of the immune system, leading to immune cells attacking the body’s own tissues. They include a wide range of systemic (e.g., systemic lupus erythematosus, rheumatoid arthritis) and organ-specific conditions (e.g., autoimmune thyroiditis), often requiring long-term immunosuppressive therapy to control inflammation and prevent organ damage [1].

Breast carcinoma is one of the most common malignancies affecting women worldwide and remains a leading cause of cancer-related morbidity and mortality. A significant proportion of cases present at a locally advanced stage, requiring multimodal treatment [2].

Although both autoimmune diseases and breast carcinoma are individually well studied, their coexistence is relatively uncommon and clinically significant. Shared genetic and immunological pathways, including chronic immune activation, regulatory T-cell dysfunction, and cytokine dysregulation, may underlie this overlap. Proposed mechanisms include tumor antigen cross-reactivity, in which immune responses directed against tumor antigens inadvertently target self-antigens, and chronic immune stimulation, which can precipitate or worsen autoimmune disease activity [3,4].

Associations have been particularly noted in patients with connective tissue diseases such as systemic lupus erythematosus, where heightened immune reactivity and immunomodulatory therapy may contribute to both cancer development and disease flare during malignancy [5].

This bidirectional interaction between malignancy and autoimmunity remains underrecognized, and formal management guidelines are lacking. We present a case of locally advanced breast carcinoma associated with a flare-up of autoimmune disease, where surgical resection of the tumor not only provided oncological control but also led to a remarkable improvement in autoimmune manifestations.

## Case presentation

**Figure 1:**
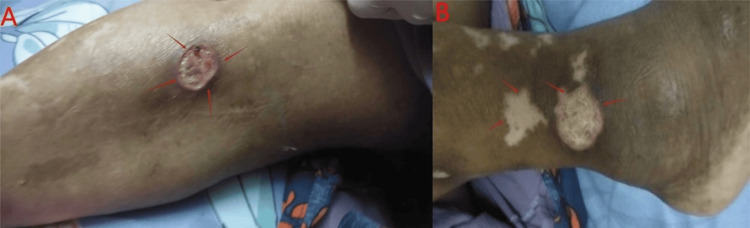
Autoimmune flare lesions in the lower limb A: ulcerative lesion with erythematous base and raised margins located over the anterior aspect of the leg (arrows); B: ulcer with surrounding hypopigmented patches suggestive of autoimmune skin involvement over the ankle region (arrows).

A 65-year-old patient presented in May 2023 with a known history of rheumatoid arthritis and systemic lupus erythematosus treated 15 years ago, with difficulty walking and multiple leg ulcers for three months (Figures 1A, 1B). Before, lupus had been in remission for several years. At presentation, pre-operative antinuclear antibody (ANA), dsDNA, and complement levels were not available, though the patient was clinically assessed for autoimmune activity.

On examination, she was partially bedridden, requiring assistance for mobility. Laboratory evaluation revealed elevated C-reactive protein (CRP) (180 IU/ml). She also had comorbidities, including type 2 diabetes mellitus (diagnosed eight years ago) and hypertension (diagnosed 10 years ago), both reasonably controlled on oral medications.

**Figure 2:**
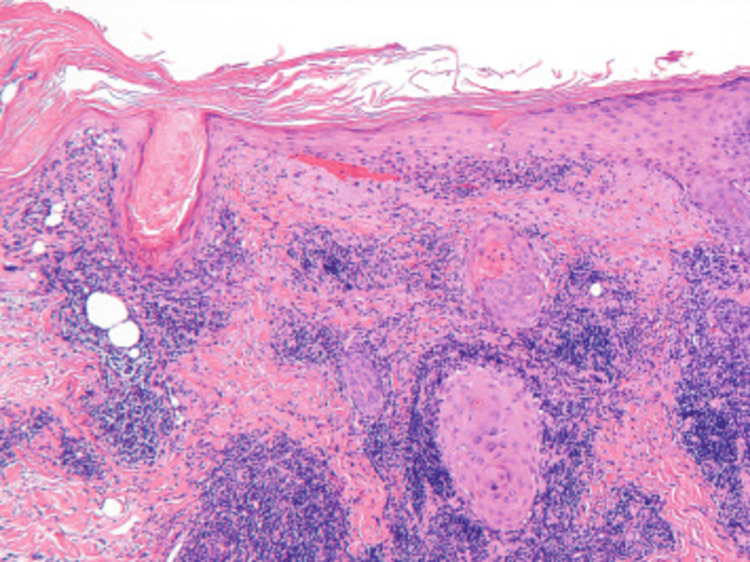
Histopathology of cutaneous lupus erythematosus (H&E stain, ×100) The epidermis shows hyperkeratosis with follicular plugging and basal cell vacuolar degeneration at the dermo-epidermal junction. Dense perivascular and periadnexal lymphocytic infiltrates are present in the dermis, consistent with cutaneous lupus erythematosus.

A skin biopsy from the ulcer margin revealed hyperkeratosis with follicular plugging, basal cell vacuolation, epidermal thinning, and dense lymphocytic infiltrate in the papillary dermis extending into subcutaneous fat, consistent with discoid lupus erythematosus (Figure 2).

**Figure 3:**
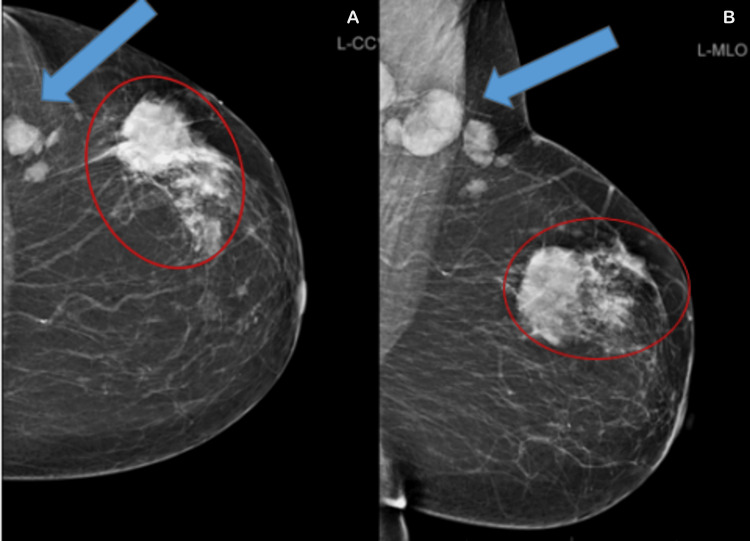
Mammographic findings A: left craniocaudal (L-CC) view showing a dominant irregular dense mass (red circle); B: left mediolateral oblique (L-MLO) view demonstrating additional suspicious nodular lesions (blue arrows) in a separate quadrant. The findings are consistent with a Breast Imaging Reporting and Data System (BI-RADS) V multi-centric malignancy.

During evaluation, a 4×3 cm mass in the left breast with multiple ipsilateral mobile axillary nodes was detected. Mammogram confirmed multicentric disease (Breast Imaging Reporting and Data System (BI-RADS) V) (Figure 3).

**Figure 4:**
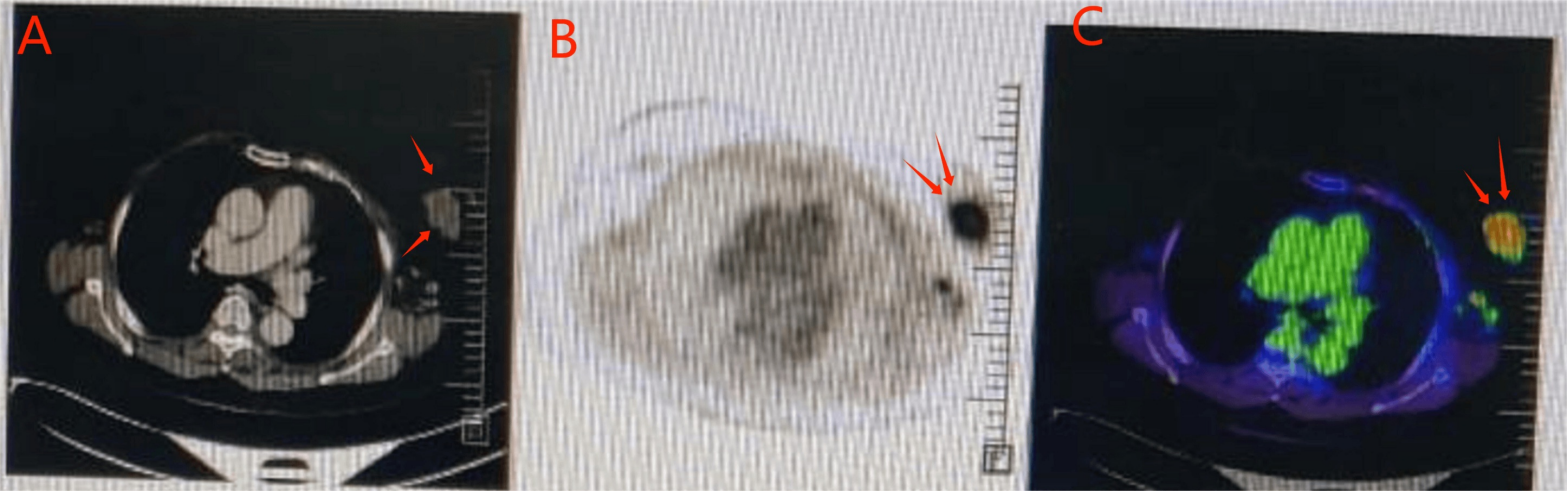
PET-CT demonstrating an FDG-avid irregular soft tissue density lesion in the left breast (arrows) with no evidence of distant metastasis A: axial CT image shows the left breast lesion; B: PET image highlights intense FDG uptake; C: fused PET-CT image confirms the metabolically active breast mass without distant lesions. FDG: fluorodeoxyglucose

Core biopsy revealed invasive ductal carcinoma Grade 2, estrogen receptor (ER)/progesterone receptor (PR) positive, human epidermal growth factor receptor 2 (HER2) negative, with high Ki67, consistent with luminal B type (Figure 4).

The PET-CT demonstrates a fluorodeoxyglucose (FDG)-avid irregular soft tissue density lesion in the left breast with no evidence of distant metastasis (Figure 4). The patient was referred to a rheumatologist, and she was started on mycophenolate mofetil (MMF) and other anti-inflammatory drugs. There was a marginal improvement in the symptoms. The patient was discussed in the multidisciplinary team, and she was started on anti-estrogen therapy. As the patient (PS=3) was in bed for more than 50% of the time and had other comorbidities, chemotherapy was deferred. The requirement for the dosage of anti-inflammatories was increasing, and the patient was not fully relieved of the autoimmune symptoms. The discussion with the rheumatologist was again sought in the multidisciplinary team after two months of anti-estrogen therapy. The rheumatologist opined that the flare-up of the autoimmune reaction appeared secondary to the malignancy, and a surgical resection would reduce the autoimmune flare. This was debated. Ultimately, a decision was made for surgery. A modified radical mastectomy was done.

**Figure 5:**
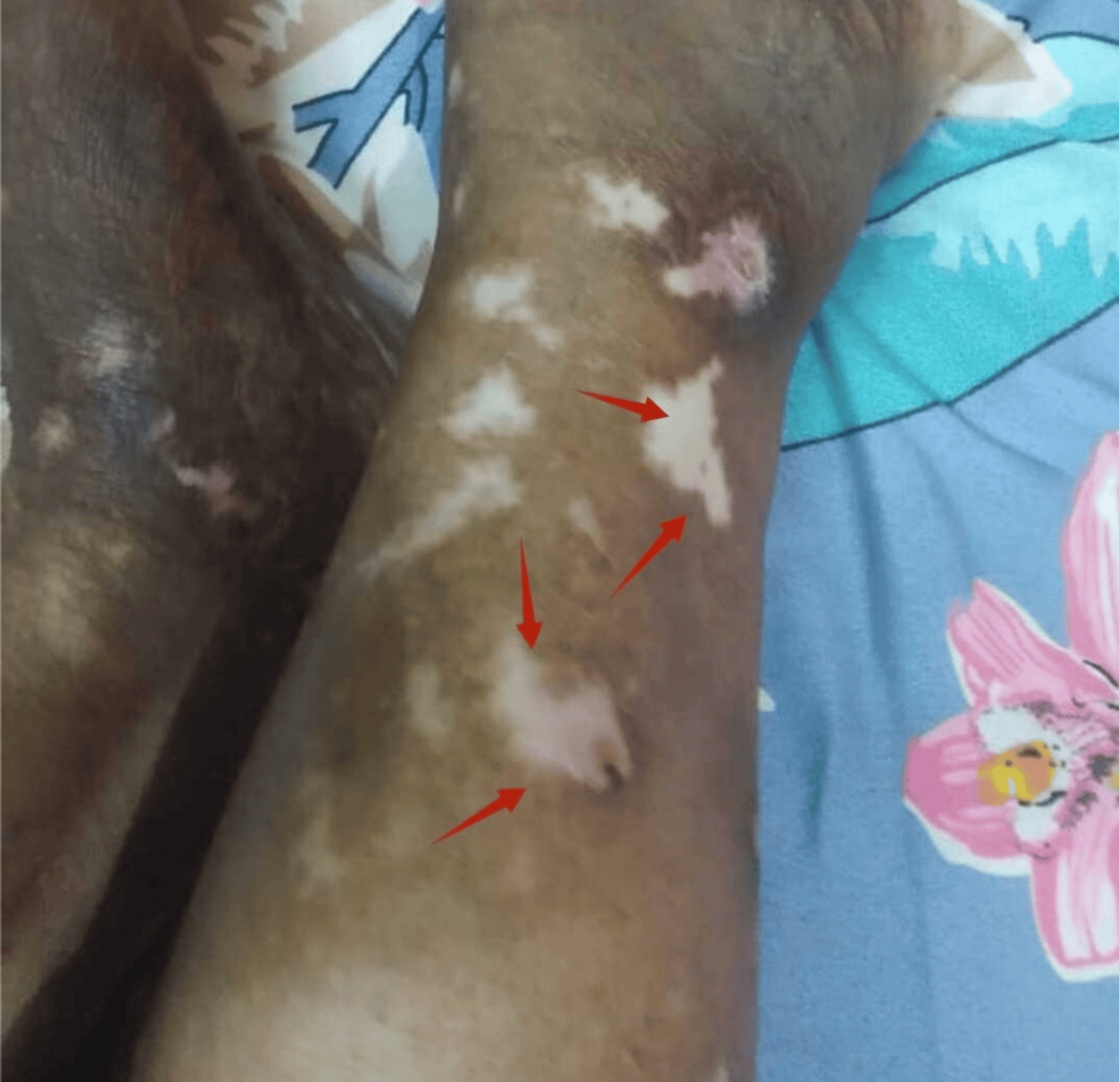
Postoperative image Arrows showing postoperative healed ulcers remission.

The post-histopathological examination was pT3N2a invasive ductal carcinoma, Grade 2, with 15 out of 20 nodes being positive. The margins were negative. Before the surgery, MMF was stopped for five days. Postoperatively, MMF was started on day 3. On the 10th post-operative day, the patient was relieved of her symptoms and was able to walk by herself without support (Figure 5). Immunosuppressive drugs were tapered and stopped.

Follow-up over 12 months confirmed sustained autoimmune remission with no recurrence of ulcers or autoimmune flare. The patient continued on adjuvant hormonal therapy in view of her ER/PR-positive, HER2-negative disease.

## Discussion

The relationship between cancer and the immune system is complex and often paradoxical. While immune mechanisms can suppress tumor growth, chronic immune activation or antigenic mimicry may conversely promote autoimmunity or even tumorigenesis [6]. Paraneoplastic autoimmunity arises when immune responses directed against tumor antigens cross-react with self-antigens or when tumor-derived cytokines disrupt peripheral tolerance [7].

In breast cancer, tumor immune-evasion pathways such as PD-L1 expression and the composition of tumor-infiltrating lymphocytes (TILs) exemplify the intricate interplay between tumor biology and host immunity [8,9]. Elevated PD-L1 levels and dense lymphocytic infiltration have been correlated with both enhanced antitumor immunity and an increased risk of autoimmune manifestations, underscoring the bidirectional influence of immune modulation in malignancy.

In our patient, the autoimmune flare persisted despite immunosuppressive therapy but subsided rapidly after definitive tumor resection, implying a tumor-driven immunologic activation. Similar outcomes have been documented in paraneoplastic myositis and pemphigus, where tumor removal led to remission of autoimmune features [10,11]. However, unlike those cases, our patient did not require prolonged immunosuppression postoperatively, suggesting that the tumor itself was the principal source of antigenic stimulation. This reinforces the concept that oncologic control can directly restore immune homeostasis by eliminating a chronic immunogenic focus.

Furthermore, autoimmune flares in cancer patients can mimic paraneoplastic syndromes, leading to diagnostic uncertainty. Population-based studies have shown increased cancer incidence in patients with dermatomyositis and polymyositis, supporting a biological link between tumor antigen expression and autoimmune activation [12]. Reports also suggest that lupus erythematosus, particularly cutaneous variants, may emerge as a paraneoplastic manifestation in breast and other epithelial cancers [13]. In our case, histopathological findings consistent with discoid lupus erythematosus (Figure 2) strengthen this association.

This case contributes to the growing evidence that paraneoplastic autoimmune phenomena may be reversible following adequate oncologic treatment. It highlights two clinically relevant insights: autoimmune exacerbations may be directly tumor-induced and resolve upon surgical resection, and timely multidisciplinary management can achieve both oncologic cure and immunologic remission. Our observation aligns with previous literature while emphasizing that complete resection can be both a therapeutic and diagnostic endpoint in tumor-associated autoimmunity.

## Conclusions

This case highlights the rare but clinically important association between breast carcinoma and autoimmune disease, where tumor presence exacerbated autoimmune activity and surgical resection led to sustained remission. The clear temporal link between tumor removal and autoimmune improvement suggests that surgical management may modulate tumor-driven immune dysregulation. Clinicians should consider underlying or progressive malignancy when encountering refractory autoimmune flares, as timely oncologic control may also restore immune balance through coordinated multidisciplinary care.
